# The associations of maternal serum ferritin levels with hypertensive disorders of pregnancy: a longitudinal cohort study

**DOI:** 10.3389/fnut.2025.1639068

**Published:** 2025-11-07

**Authors:** Huiqin Mo, Zijun Wang, Cuicui Qu, Xiaohua Liu

**Affiliations:** 1 Department of Obstetrics, Shanghai Key Laboratory of Maternal and Fetal Medicine, Obstetrics and Gynecology Hospital, Tongji University School of Medicine, Shanghai, China; 2 Department of Obstetrics and Gynecology, The Seventh People's Hospital of Shanghai University of Traditional Chinese Medicine, Shanghai, China

**Keywords:** hypertensive disorders of pregnancy, iron supplementation, longitudinal changes, pregnant women, serum ferritin

## Abstract

**Background:**

The longitudinal fluctuations in maternal serum ferritin (SF) levels during gestation, acting as indirect indicators of iron supplementation, have not been thoroughly investigated in relation to the incidence of hypertensive disorders of pregnancy (HDP).

**Methods:**

A retrospective cohort investigation was carried out at a tertiary maternity hospital in Shanghai, involving women with serum ferritin (SF) measurements at 8.0–13.6 gestational weeks (GW) and at 29.0–31.6 GW. Logistic regression analysis was employed to evaluate the relationship between maternal SF levels, their longitudinal variations, with the risk of HDP.

**Results:**

The study included 17,472 women, among whom 473(2.71%) developed gestational hypertension (GH) and 560(3.21%) developed preeclampsia (PE). Adjusted odds ratios (ORs; 95% confidence intervals) for HDP across ascending quartiles of SF concentrations were as follows: at 8.0–13.6 GW, 1.00 (reference), 1.043 (0.864–1.258), 1.060 (0.878–1.279), and 1.234 (1.027–1.482); and at 29.0–31.6 GW, 1.00 (reference), 0.973 (0.800–1.181), 1.076 (0.890–1.301), and 1.299 (1.082–1.560). Women with SF levels in the highest quartile at 8.0–13.6 GW exhibited reduced HDP risk when their SF levels declined to the lowest quartile by 29.0–31.6 GW. Conversely, those with SF levels in the lowest quartile early in pregnancy but transitioning to the highest quartile later in pregnancy demonstrated a significantly elevated HDP incidence (8.2%; OR: 1.445, 95% CI: 1.003–2.081).

**Conclusion:**

Maternal SF levels demonstrated an independent positive association with HDP risk during early and late gestational stages. These findings suggest that routine iron supplementation in iron-replete women may exacerbate HDP risk and warrants careful reconsideration.

## Introduction

Hypertensive disorders of pregnancy (HDP) encompass gestational hypertension (GH), preeclampsia (PE)-eclampsia, chronic hypertension, and preeclampsia superimposed on chronic hypertension (1). GH and PE-eclampsia are classically characterized by the emergence of new-onset hypertension after 20 weeks of gestation, which are key subtypes responsible for maternal and prenatal morbidity and mortality (2, 3). Although the exact etiology of HDP remains uncertain, current evidence indicates that these conditions are primarily placenta-mediated. Key contributors such as oxidative stress (4), immune tolerance (5), defective deep placentation and endothelial dysfunction are believed to play crucial roles. Recently, several studies indicates that the pathogenesis of preeclampsia may stem from iron-induced oxidative stress and endothelial dysfunction (6–8).

Prenatal iron supplementation is a proven strategy for averting maternal iron deficiency anemia (IDA) throughout pregnancy. The World Health Organization (WHO) advocates a daily iron supplement dosage ranging between 30 and 60 mg for pregnant individuals because of the high incidence of IDA. Additionally, WHO recommends the assessment of serum ferritin (SF) levels as the optimal screening method for evaluating iron stores in standard clinical practice (9, 10). Nevertheless, caution is warranted when implementing iron supplementation due to the potential for changes in iron levels that may signal bodily accumulation. This accumulation can trigger the biochemical generation of reactive oxygen species, leading to the abnormal degration of proteins, DNA, and mitochondria (6, 11). Previous epidemiological findings have highlighted significant concerns regarding the co-occurrence of elevated serum ferritin levels with adverse pregnancy outcomes during pregnancy (12–14). In a prior cohort study, we examined the longitudinal fluctuations in maternal plasma ferritin levels, revealing a positive correlation with the risk of gestational diabetes mellitus (GDM) during both the early and late trimesters (15).

While HDP have been linked to elevated SF levels (16–18), the effects of iron supplementation on pregnant individuals remain an ongoing area of investigation. The conventional approach of iron supplementation may pose risks to maternal and perinatal well-being in certain iron-replete women, emphasizing the necessity of monitoring ferritin levels throughout pregnancy to tailor iron supplementation strategies. Therefore, we undertook a longitudinal cohort study to evaluate the correlation between maternal SF levels (across the entire range) during early and late gestational periods and the incidence of HDP, as well as to investigate the impact of longitudinal changes in maternal SF levels on the development of HDP.

## Methods

### Data source and study participants

This retrospective cohort study was carried out on pregnant women registered at Shanghai First Maternity and Infant Hospital (SFMIH), Tongji University School of Medicine, spanning the period from May 30, 2018 to December 22, 2020. The study included individuals who underwent SF concentration evaluations at gestational weeks 8.0–13.6 and 29.0–31.6. Following the exclusion of women with pre-existing chronic hypertension (identified before 20 weeks of gestation, including primary, cardiogenic or nephrogenic hypertension), multiple gestation, chronic infections, thalassemia and autoimmune diseases, a total of 17,472 women met the inclusion criteria and were analyzed. Supplementary Figure S1 illustrates the study population’s flowchart.

Data were extracted from the hospital’s electronic medical records systems, including information on prenatal fertility-related characteristics, obstetric complications, labor and delivery summaries, and postpartum maternal and neonatal information. The prenatal information of the participants was obtained through a routine baseline questionnaire during the first prenatal visit (8.0–13.6 GW), which included blood pressure, maternal age, educational level, gravidity, parity, and medical history and medications. In subsequent prenatal visits, registered pregnant women will be followed by serum ferritin level testing again between 29.0–31.6 GW. Pre-pregnancy body mass index (BMI) was calculated based on self-reported pre-pregnancy weight and height. Gestational weeks were estimated based on self-reported last menstrual period and confirmed by first-trimester ultrasound.

PE and GH were diagnosed according to the guidelines from The American College of Obstetricians and Gynecologists (19). GH is new-onset hypertension (systolic blood pressure [SBP] ≥ 140 mmHg or diastolic blood pressure [DBP] ≥ 90 mmHg on two occasions at least 4 h) after gestational week 20, no evidence of proteinuria (< 300 mg in 24 h). Based on these criteria, pregnancies displaying proteinuria or featuring new-onset symptoms such as thrombocytopenia, renal insufficiency, impaired liver function, pulmonary edema, cerebral, or visual disturbances were diagnosed as PE.

SF levels were assessed employing a commercially available Maglumi ferritin immunoluminometric assay conducted on a Beckman Coulter Dxl 800 access instrument at our institution. The reagents and calibrants utilized originated from the identical manufacturer, with the interassay coefficient of variation for this evaluation was < 5% (20, 21). All laboratory data are routinely validated through internal quality assurance programs and processed according to standardized analytical procedures.

### Statistical analysis

The data were assessed for missing values, outliers, and logical consistency. Continuous variables were expressed as mean ± standard error (SE) for normally distributed data or median (range) for non-normally distributed data, while categorical variables were summarized as frequencies and percentages. Statistical analyses were performed using appropriate tests based on data distribution: chi-square tests for categorical variables, independent t-tests for normally distributed continuous variables between two groups, one-way ANOVA for multiple group comparisons of normally distributed data, and Kruskal-Wallis tests for non-normally distributed continuous variables. SF concentrations and blood sampling were measured at 8.0–13.6 GW and 29.0–31.6 GW. Quartiles of SF concentrations were established separately for each trimester. Initial analysis categorized women into quartiles (Q1-Q4) to evaluate the two different trimester SF-HDP associations. Participants meeting both criteria were excluded to minimize confounding by chronic inflammation: elevated SF (>100 μg/L, suggesting inflammation) and anemia (Hb < 110 g/L). For longitudinal assessment, participants were stratified into 9 subgroups based on SF concentration changes between trimesters, defined as low (< 25th percentile), intermediate (25th-75th percentile), and high (> 75th percentile) at each time point, creating a 3 × 3 matrix with the intermediate-intermediate subgroup as reference. Associations were evaluated using logistic regression to calculate odds ratios (ORs) with 95% confidence intervals (CIs). Variance inflation factor (VIF) was used to test the multicollinearity. Results indicated that there were no problem of serious multicollinearity in this study (VIFs 1.00–1.30, all VIFs < 2.0 indicating no significant multicollinearity). Multivariable models adjusted for potential confounders, including age, pre-pregnancy BMI, GDM, Intrahepatic Cholestasis of Pregnancy (ICP), parity, white cell count, and gestational weeks. Tests for trends were conducted by assigning median SF values to each quartile as continuous variables. Given the non-normality of SF distribution, restricted cubic splines (RCS) with 5 knots (5th, 27.5th, 50th, 72.5th, and 95th) were implemented in the Stata (version 17.0; Stata Corp) to model non-linear relationships between continuous SF levels and HDP risk, with covariate adjustment. We compared models with 4 to 6 knots and found minimal differences in the Akaike Information Criterion (AIC), with ΔAIC < 2, indicating consistent results across knot specifications. The overall *p* value was calculated from the basic Poisson regression model in generalized linear modeling, while the nonlinear p value was derived from the Poisson regression model incorporating nonlinear terms RCS-transformed SF terms (knots = 5). Additional analyses were performed using SPSS (version 26; IBM Corp), and key regression results were re-validated using RStudio (version 4.3.3; Posit Software, PBC). Multiple comparisons using the Bonferroni correction, a two-tailed *p*-value <0.05 defined statistical significance.

To reduce heterogeneity, subgroup analyses were conducted between covariates: age (< 35 years old vs. ≥ 35 years old), GDM (yes/no), preterm (yes/no), BMI (< 28 vs. ≥ 28 kg/m^2^), parity (< 3 vs. ≥ 3), ICP (yes/no), PROM (yes/no). And interaction effects were tested by conducting likelihood ratio tests comparing the main regression analysis with the interaction in Supplementary Table S1, with no significant interactions detected (all P for interaction > 0.20). Furthermore, we performed sensitivity analyses to examine the robustness of our findings via the Receiver Operating Characteristic (ROC) curves.

## Results

The baseline characteristics of the control group, women with PE, and with GH are detailed in Table 1. In our 17,472 pregnant women cohort, 473 cases (2.71%) were diagnosed with GH, and 560 cases (3.21%) were diagnosed with PE. Women with GH and PE exhibited older age, higher body weight, elevated incidence of complications such as gestational diabetes (GDM), and a reduced propensity for premature rupture of membranes (PROM; Table 1). Elevated serum ferritin levels were notably observed in the GH and PE groups at the two measurement times. Furthermore, for the GH women, the proportion of preterm birth and small for gestational age (SGA) were the lowest. However, there were more cases of ICP and SGA in the PE women, but a decreased vaginal delivery rate. Hemoglobin (Hb) levels and white blood cell (WBC) count in the GH and PE group were both higher at the two measurement times. Besides, the proportion of multiparity ≥ 3, and neonatal asphyxia (Apgar scores ≤ 7) did not display significant difference among the three groups.

**Table 1:** The baseline characteristics of participants.

Characteristics	Overall (*N* = 17,472)	Control group (*N* = 16,439)	GH (*N* = 473)	PE (*N* = 560)	*p*
Age(y)^a^	31.26 ± 0.03	31.21 ± 0.03	32.40 ± 0.19	31.60 ± 0.17	**<0.001**
Parity≥3 (n, %)	157(0.9)	149(0.9)	5(1.1)	3(0.5)	0.615
BMI (kg/m^2^)^a^	22.25 ± 0.18	22.06 ± 0.19	24.92 ± 0.69	25.48 ± 1.25	**<0.001**
BMI ≥ 28 (n, %)	611(3.5)	466(2.8)	75(15.9)	70(12.5)	**<0.001**
LGA (n, %)	808(4.6)	757(4.6)	20(4.2)	31(5.5)	0.539
SGA (n, %)	537(3.1)	479(2.9)	11(2.3)	47(8.3)	**<0.001**
GDM (n, %)	2,147(12.3)	1960(11.9)	89(18.8)	98(17.5)	**<0.001**
ICP (n, %)	202(1.2)	183(1.1)	5(1.1)	14(2.5)	0.010
PROM (n, %)	3,037(17.4)	2,928(17.8)	61(12.9)	48(8.6)	**<0.001**
Preterm (n, %)	762(4.4)	661(4.0)	16(3.4)	85(15.2)	**<0.001**
Vaginal delivery (n, %)	9,524(54.5)	9,157(55.7)	189(40.0)	178(31.8)	**<0.001**
Gestational week^a^	39.12 ± 0.01	39.2 ± 0.01	38.90 ± 0.05	38.37 ± 0.08	**<0.001**
Apgar scores≤7, (n, %)	145(0.8)	132(0.8)	4(0.8)	9(1.6)	0.119
SF at first collection, μg/L^a^	62.92 ± 0.35	62.68 ± 0.36	68.89 ± 2.59	64.88 ± 1.99	0.010
SF at second collection, μg/L^a^	14.39 ± 0.09	14.21 ± 0.09	17.08 ± 0.63	17.39 ± 0.81	**<0.001**
Gestational week at first collection, week^b^	11.2(8.0–13.6)	11.2(8.0–13.6)	11.2(8.0–13.6)	11.2(8.0–13.6)	0.899
Gestational week at second collection, week^b^	31.1(29.0–31.6)	31.1(29.0–31.6)	30.6(29.0–31.6)	31.0(29.0–31.6)	**<0.001**
Hemoglobin at first collection, g/L^a^	126.88 ± 0.07	126.69 ± 0.07	129.98 ± 0.42	129.86 ± 0.41	**<0.001**
Hemoglobin at second collection, g/L^a^	115.44 ± 0.07	115.24 ± 0.07	118.30 ± 0.46	118.88 ± 0.45	**<0.001**
WBC count at first collection, 10^^9^/L^a^	8.35 ± 0.01	8.30 ± 0.01	9.15 ± 0.10	9.01 ± 0.10	**<0.001**
WBC count at second collection, 10^^9^/L^a^	9.11 ± 0.02	9.09 ± 0.02	9.56 ± 0.10	9.51 ± 0.10	**<0.001**

a Values are means ± SEs(standard errors).

b Values are medians(minimum value-maximum value), and else are proportions for categorical variables. Categorical variables were analyzed using the Kruskal-Wallis test, while continuous variables were analyzed using the one-way analysis of variance (ANOVA).

BMI, body mass index; LGA, large for gestational age; SGA, small for gestational age; ICP, intrahepatic cholestasis of pregnancy; PROM, premature rupture of membranes; SF, serum ferritin; WBC, white blood cell. Bold values indicate statistical significance at the *p* < 0.05 level.

The median concentration of ferritin in our study cohort decreased significantly from 51.7 μg/L (IQR: 31.5–81.9 μg/L) at 8.0–13.6 GW to 11.3 μg/L (IQR: 7.5–18.1 μg/L) at 29.0–31.6 GW. Table 2 shows the risk of HDP correlated with distinct quartiles of SF among pregnant women. A elevated risk of HDP was noted in women with escalation in SF quartiles during both trimesters, with individuals in the highest SF quartile (quartile 4, upper 25%) experiencing the highest susceptibility to HDP. The unadjusted ORs of HDP increased with ascending quartiles of ferritin at 8.0–13.6 GW (p for trend = 0.084) and at 29.0–31.6 GW (p for trend < 0.001). After adjusting for maternal age, BMI, gestational week, ICP, GDM, PROM and preterm, the adjusted OR (95% CI) for HDP in the highest quartile of ferritin at 8.0–13.6 GW was 1.234(1.027, 1.482). Furthermore, the adjusted OR (95% CI) of HDP in the highest quartile of ferritin was 1.299(1.082, 1.560) at 29.0–31.6 GW, which is similar to the trend at 8.0–13.6 GW. Although consistent associations observed in both early and late gestational periods, the highest quartile (Q4) of SF levels at 29.0–31.6 GW demonstrated the greatest risk of HDP, with a prevalence of 7.2% in this subgroup(adjusted OR = 1.299, 95% CI: 1.082–1.560).

**Table 2:** Association between quartiles of SF and the risk of HDP.

Variables	HDP (GH + PE, n, %)	Unadjusted^a^ OR(95%CI)	Adjusted^b^ OR(95%CI)
Quartiles of SF with HDP risk in early pregnancy			
Quartile 1	250(5.7)	1(ref)	1(ref)
Quartile 2	246(5.6)	0.995(0.830–1.193)	1.043(0.864–1.258)
Quartile 3	253(5.8)	1.021(0.852–1.222)	1.060(0.878–1.279)
Quartile 4	284(6.5)	1.165(0.978–1.389)	1.234(1.027–1.482)
P for trend^c^		0.084	**0.028**
Quartiles of SF with HDP risk in late pregnancy			
Quartile 1	238(5.3)	1(ref)	1(ref)
Quartile 2	232(5.4)	1.028(0.854–1.238)	0.973(0.800–1.181)
Quartile 3	249(5.7)	1.086(0.905–1.304)	1.076(0.890–1.301)
Quartile 4	314(7.2)	1.400(1.177–1.666)	1.299(1.082–1.560)
P for trend^c^		**<0.001**	**0.002**

a Odds ratios (ORs) and their 95% confidence intervals (CIs) were estimated using multivariable logistic regression.

b Logistic regression adjusted for maternal age(continuous), BMI(continuous), gestational week(continuous), ICP (yes/no), GDM(yes/no), PROM(yes/no) and preterm(yes/no).

c P for trends were performed by fitting median values for each quartile of SF concentrations as a continuous variable.

Bold values indicate statistical significance at the *p* < 0.05 level.

Table 3 shows the association of longitudinal changes in SF concentrations across two different trimesters and the incidence of HDP. Women in the quartiles 2 and 3 of ferritin concentrations during both trimesters were combined as the reference group, denoted as the 2×2 group (comprising pregnant individuals with SF levels ranging from 31.5–81.9 μg/L at 8.0–13.6 GW and 7.5–18.1 μg/L at 29.0–31.6 GW). Noteworthy findings from the forest plot revealed a distinct discontinuity in HDP risk. Specifically, the subset of women identified with quartile 4 at 8.0–13.6 GW but decreased to quantile 1 at 29.0–31.6 GW having the lowest incidence of HDP (3.5%, 0.697[0.402–1.209]), while the incidence increased to 8.2% (1.445[1.003–2.081]) if the quartile 1 at first measurement ascended to quartile 4 at second measurement. Furthermore, individuals in the quartile 4 category at both 8.0–13.6 GW and 29.0–31.6 GW exhibited a statistically significant correlation with the incidence of HDP (adjusted OR = 1.339, 95% CI: 1.076–1.667, *p* = 0.009).

**Table 3:** The combined effect of serum ferritin concentrations during two different trimesters on HDP.

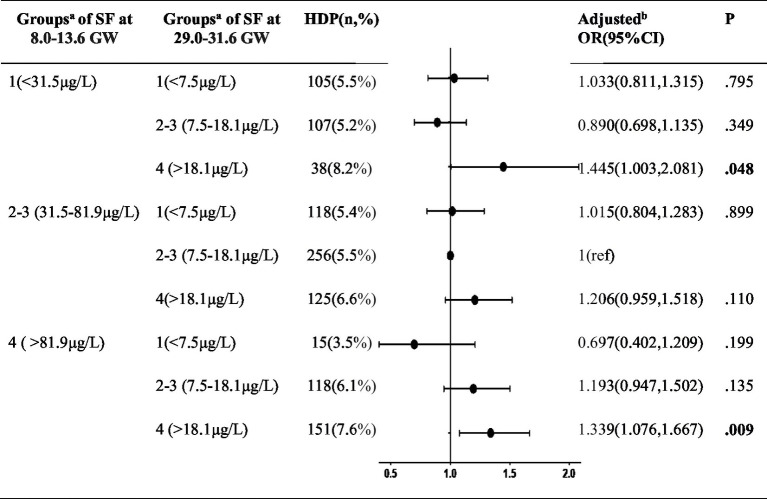

^a^Pregnant women were stratified into 9 groups based on quartiles of SF concentrations at two measurements (low, <25 percentile; intermediate, 25 ~ 75 percentile; and high, >75 percentile in two measurements). This created a 3 × 3 matrix, with the intermediate group from both measurements (25th-75th percentile in both assessments) serving as the reference group. ^b^Logistic regression adjusted for maternal age(continuous), BMI(continuous) and ICP (yes/no), GDM(yes/no), PROM(yes/no) and Preterm(yes/no).

**Figure 1:**
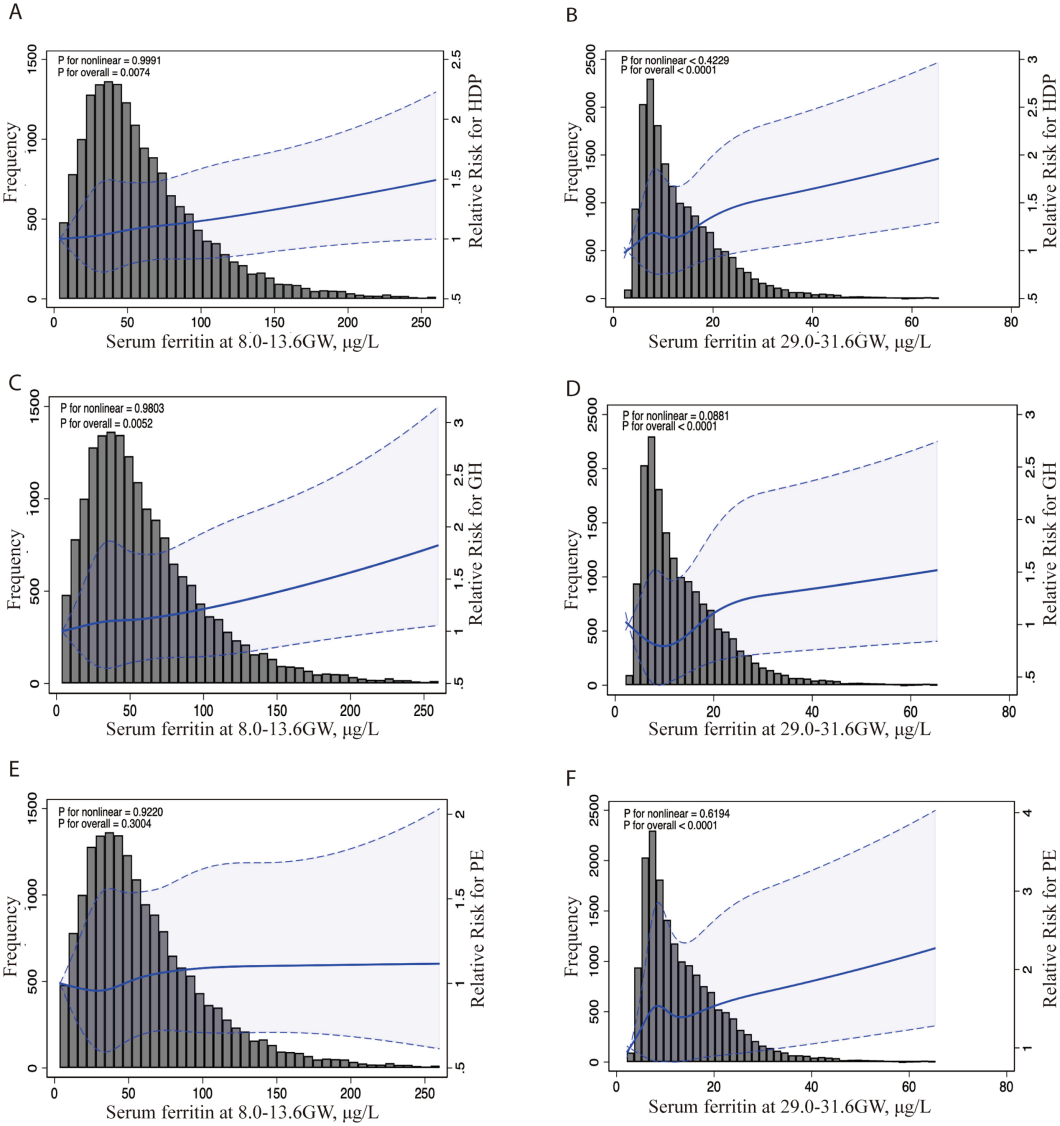
Association between serum ferritin levels in two different trimesters and the risk of HDP, GH, and PE. Legend: The RCS curves depict adjusted ORs and 95% CIs for the association between serum ferritin levels at 8.0–13.6 GW and the risk of HDP **(A)**, GH **(C)**, and PE **(E)**. Similarly **(B,D,F)** show the associations at 29.0–31.6 GW with HDP, GH, and PE risk. The histogram displays the distribution of participants by SF concentration levels. The solid line indicates the point estimates of ORs, while the dotted lines represent the 95% CIs. These ORs were derived from a restricted cubic spline log-Poisson regression model, with adjustments for maternal age (continuous), BMI (continuous) and ICP (yes/no), GDM (yes/no), PROM (yes/no) and preterm (yes/no). HDP, hypertensive disorders of pregnancy; GH, gestational hypertension; PE, preeclampsia (PE)-eclampsia; RCS, restricted cubic splines; OR, odds ratios.

**Figure 2:**
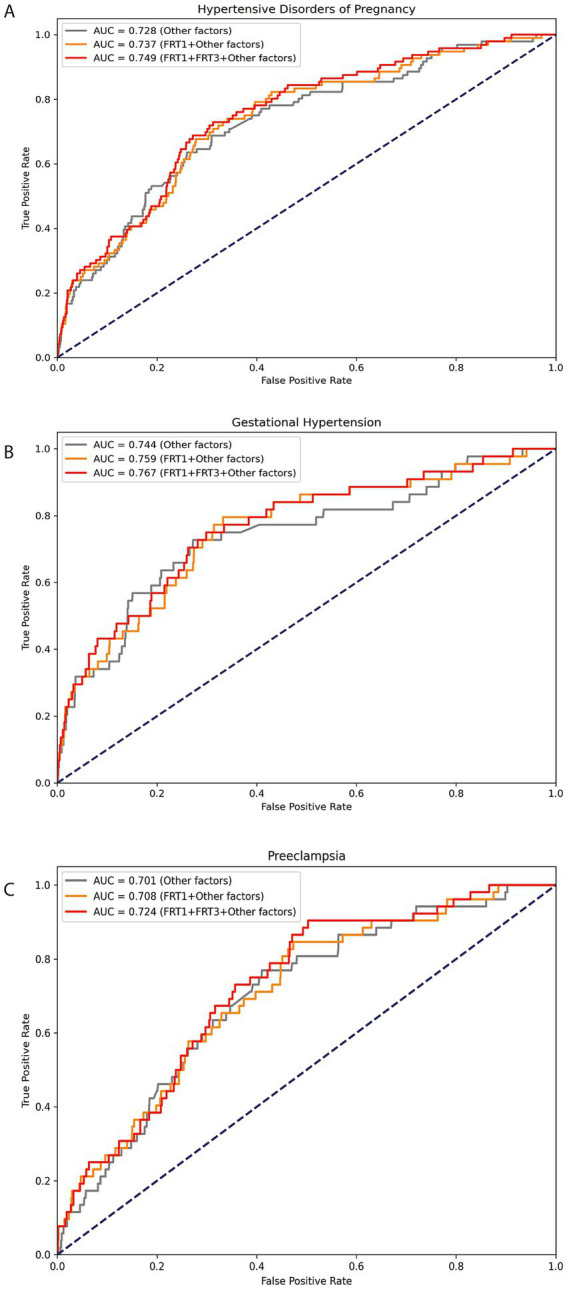
ROC analysis of serum ferritin for predicting HDP/GH/PE. Legend: The ROC curves depict the predictive performance of serum ferritin for HDP **(A)**, GH **(B)**, and PE **(C)**. FRT1: serum ferritin levels at 8.0–13.6 GW; FRT3: serum ferritin levels at 29.0–31.6 GW; Other factors: maternal age (continuous), BMI (continuous) and ICP (yes/no), GDM (yes/no), PROM (yes/no), ICP (yes/no) and preterm (yes/no).

To assessment the continuous association between the SF levels and the risk HDP (including GH and PE) at 8.0–13.6 GW and 29.0–31.6 GW, we used the restricted cubic splines (RCS) in Figure 1. The graph visually illustrates the increasing HDP risk as SF levels increases during two gestational periods after adjusting for age, pre-pregnancy BMI, GDM, ICP, preterm and parity at each testing. Additionally, we constructed ROC curves to assess the predictive performance of our models, as shown in Figure 2. The combined model integrating serum ferritin levels from both 8.0–13.6 and 29.0–31.6 GW with clinical confounders demonstrated the highest predictive performance, indicating that serum ferritin provides incremental value over established factors and serves as a complementary tool for risk stratification.

## Discussion

Our longitude cohort study revealed the association between the longitudinal variations in SF levels and HDP risk with a large sample size. Four key findings had yielded: (1) Maternal SF levels demonstrate a noticeable decrease from early to late gestational stages. (2) Maternal SF levels exhibit a marked independent positive correlation with HDP risk throughout both early and late gestational periods. (3) Elevated SF levels in early pregnancy did not increase the risk of PE while later elevated SF levels increased the risk. (4) The risk of HDP associated with elevated SF levels in early pregnancy was normalized if these levels subsequently decrease to a lower range in late pregnancy.

Ferritin is a well-established biomarker for evaluating iron status, differentiating IDA from other microcytic anemias, and identifying iron overload (22, 23). Our longitudinal findings demonstrate a significant gestational decline in both ferritin and hemoglobin levels, consistent with the majority of established research (15, 24). This physiological reduction primarily reflects increased iron demands for the feto-placental development, elevated maternal erythrocyte mass, and maternal plasma volume expansion during pregnancy (25, 26).

In nonpregnant populations, the relationship between SF and hypertension remains inconsistent. While some studies have highlighted serum ferritin as a critical predictor of hypertension in middle-aged Korean men (27, 28), others have reported divergent findings. Zhu et al. observed a positive correlation between hemoglobin and transferrin with blood pressure but found no significant correlation between ferritin or soluble transferrin receptor (sTFR) levels and blood pressure (29). Notably, a cross-sectional study demonstrated distinct associations between ferritin and blood pressure: a positive linear correlation with DBP in nonpregnant women and an inverted U-shaped relationship with SBP during pregnancy (30). In pregnant populations, previous studies have reported iron overload in individuals with hypertensive disorders of pregnancy, characterized by elevated serum iron, ferritin, and transferrin saturation levels (31, 32). In a case–control study on preeclamptic patients between 32 and 38 gestational weeks reported a positive correlation between SF levels and DBP (18). However, Taeubert et al. found no associations between maternal iron status in early pregnancy and maternal blood pressure or placental hemodynamics (33). The inconsistency in these findings may be attributed to the dynamic nature of iron stores during pregnancy, as most studies have focused on iron status at specific gestational stages. To investigate this relationship in a Chinese longitudinal cohort, we analyzed electronic medical records from 17,472 pregnancies. Recently, a retrospective cohort study investigated SF levels in early pregnancy (<12 GW) and late pregnancy (>28 GW), and suggested that the association between SF levels in early pregnancy with HDP was stronger than that in late pregnancy (34). Our findings demonstrate a positive correlation between ferritin levels and the risk of HDP in both early and late gestational periods. Furthermore, we investigate the longitudinal variation in SF levels from early to late gestational phases in relation to the risk of HDP. We found that the elevated risk of HDP associated with high SF levels in early pregnancy diminishes if SF levels decline to a lower levels in late pregnancy.

Routine iron supplementation during pregnancy has been demonstrated to significantly decrease the prevalence of maternal anemia at delivery (35, 36). Current clinical guidelines recommend iron supplementation for reproductive-aged women with SF levels <15 μg/L, indicating potential iron deficiency or IDA, while those maintaining SF levels >70 μg/L are generally considered sufficient in iron stores and do not require supplementation (37). Current guidelines and studies vary in defining iron deficiency ferritin thresholds in pregnancy, WHO recommends a ferritin cut-off value of <15 μg/L in the first trimester, and the United States use a threshold of 10–15 μg/L, while the United Kingdom and some other countries use <30 μg/L (9, 35, 38, 39). Based on a nationwide cross-sectional study, Tan et al. reported that the prevalence of IDA increased across pregnancy trimesters, peaking during the eighth month (40). Our findings reveal significant trimester-specific variations in iron status among pregnant women. While only 23.1% of participants exhibited SF levels < 30 μg/L during the first trimester, this proportion dramatically increased to 93.8% by the third trimester. Concurrently, 25.4% of pregnant women demonstrated Hb levels <110 g/L at 29.0–31.6 GW in our study, meeting the diagnostic criteria for anemia. These data suggest that the current clinical threshold of SF < 30 μg/L (41), commonly used in Chinese obstetric practice, lacks sufficient sensitivity for guiding iron supplementation in late pregnancy. The progressive decline in ferritin concentrations throughout gestation underscores the necessity of establishing trimester-specific reference ranges, rather than relying on a uniform cutoff value, to accurately assess maternal iron stores and guide iron supplementation across different trimesters.

As an established indicator of iron status, serum ferritin provides reliable measurement of both iron deficiency and excess. Excessive maternal iron stores could potentially elevate blood pressure during gestation and impair uteroplacental vascular function through oxidative stress-mediated pathways (4). Recent investigations into the pathogenesis of HDP have increasingly focused on ferroptosis, an iron-dependent form of programmed cell death (42–44). Early in gestation, extravillous trophoblasts (EVTs) invade decidual tissues and spiral arteries, processes that elevates oxygen and iron concentrations in the placenta. This iron overload facilitates Reactive Oxygen Species production via Fenton reactions, thereby inducing excessive lipid peroxidation and ferroptotic cell death in trophoblasts. The observed Perls’-positive iron deposits in placental tissue suggest iron overload in HDP (31, 45). Such pathological changes impair EVT invasion, disrupt spiral artery remodeling, and induce vasoconstriction, ultimately contributing to the development of preeclampsia (8, 30, 46). A physiological decline in SF concentrations is observed throughout gestation, with minimal levels typically attained during late pregnancy (40, 47). Beyond hemodilution effects, the ferritin decrease likely represents physiological iron store mobilization, a process facilitated by the characteristic gestational decline in hepcidin concentrations. Hepcidin, a key regulator of iron metabolism, plays a critical role in protecting cells from iron-mediated cytotoxicity. Reduced hepcidin levels and elevated plasma iron concentrations have been associated with the pathogenesis of preeclampsia (7, 48). Our findings suggest a correlation between elevated SF levels and the risk of HDP. Furthermore, we excluded 19 cases of HDP with delivery prior to 34 GW and subsequently repeated our analyses as shown in Supplementary Table S2. The results remained consistent with our primary findings. However, elevated serum ferritin may indicate not only increased maternal iron stores but also underlying inflammation in pathological pregnancies or impaired plasma volume expansion. Accordingly, causality remains unestablished, necessitating further investigation into the underlying mechanisms.

Our study has several limitations that warrant consideration. Firstly, while SF serves as a widely adopted biomarker for iron status assessment, its diagnostic reliability is constrained by its acute-phase reactant properties, particularly during inflammatory conditions. To mitigate this limitation, we excluded participants exhibiting anemia of inflammation, defined by concurrent SF > 100 μg/L and Hb < 110 g/L. Furthermore, we adjusted for key confounding variables, including maternal age, pre-pregnancy BMI, GDM, ICP, and preterm. However, the retrospective design inherently limited our ability to incorporate additional clinically relevant data, such as longitudinal blood pressure measurements, HDP diagnosis/duration, antihypertensive use, socioeconomic status, and smoking history. Other important iron metabolism markers (e.g., sTfR, serum iron and hepcidin), along with IL-6, C-reactive protein, and uric acid, may improve the interpretation of our results (48, 49). Future prospective studies incorporating comprehensive biochemical data and longitudinal monitoring are needed to validate and extend our findings. Thirdly, due to the retrospective nature of our study, we could not obtain precise data on iron supplementation, including dosage and duration. Therefore, our assessment of supplementation efficacy was indirectly replaced by longitudinal changes in maternal SF levels throughout pregnancy. In our study, we combined PE and GH into HDP group for analysis. The RCS curves showed no significant association between SF during the first trimester and PE risk, whereas third-trimester SF elevations were strongly predictive of PE. The discrepant results related to SF may be attributed to differences in the underlying etiological mechanisms of PE and GH, with severe PE potentially being a placental disease, while GH is more likely a maternal condition (50). Further studies are needed to elucidate the distinct mechanisms involved.

In conclusion, our study reveals a significant positive association between elevated SF levels and the risk of HDP, underscoring the importance of monitoring iron status during gestation. For pregnant, routine iron supplementation may not be advisable for iron-replete women or those at high risk of HDP, given the potential association with increased HDP risk.

## Data Availability

The raw data supporting the conclusions of this article will be made available by the authors, without undue reservation.

## Supplementary material

SUPPLEMENTARY FIGURE S1 Flowchart of the study population.
