# Supplementary figures and images for: The associations of maternal serum ferritin levels with hypertensive disorders of pregnancy: a longitudinal cohort study
Source: Front Nutr. 2025 Nov 7;12:1639068. doi: 10.3389/fnut.2025.1639068 (PMC12637025; doi:10.3389/fnut.2025.1639068)

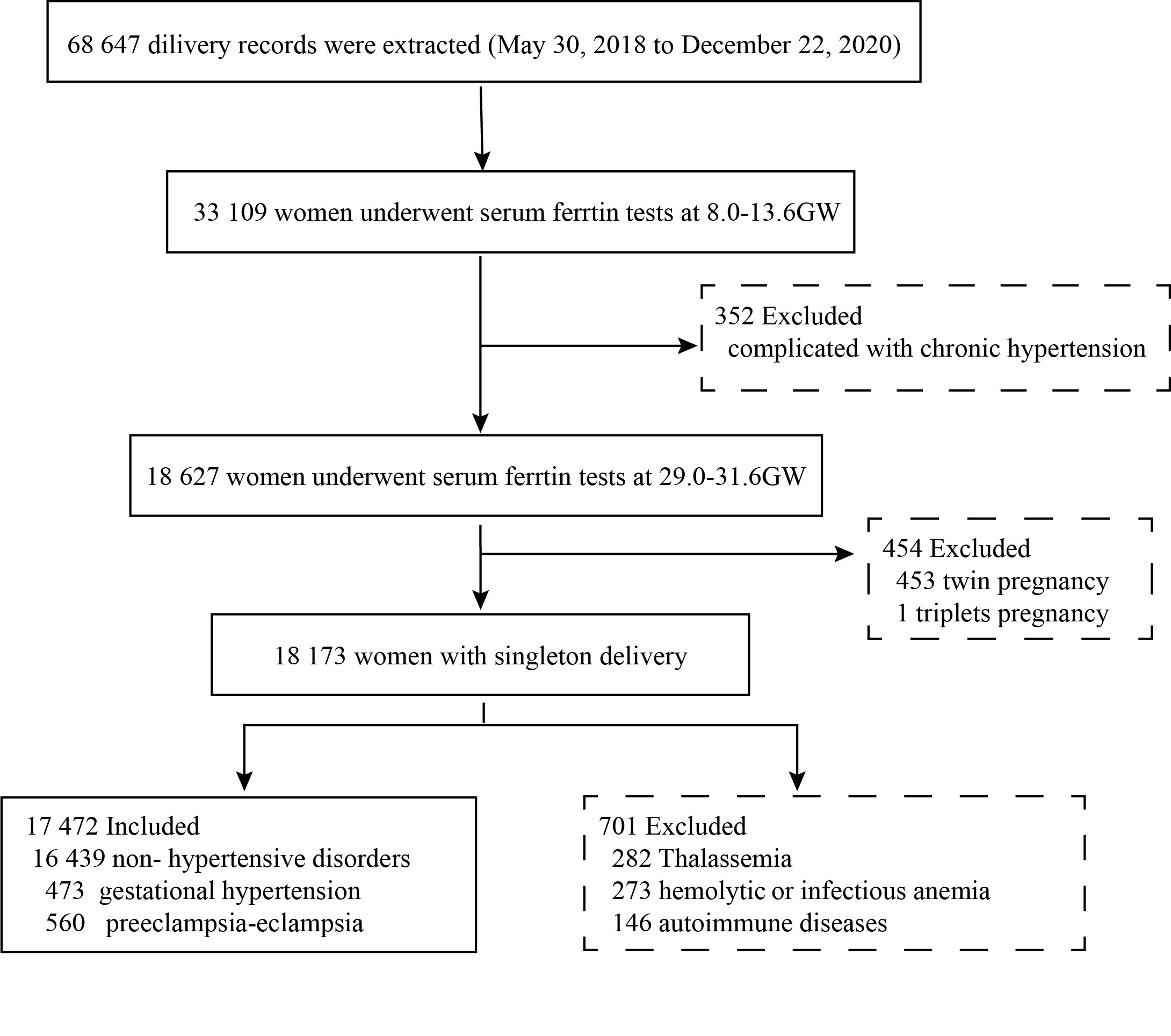

Supplement: SUPPLEMENTARY FIGURE S1 — Flowchart of the study population. [file Image_1.JPEG]
