# Supplementary material for: The associations of maternal serum ferritin levels with hypertensive disorders of pregnancy: a longitudinal cohort study
Source: Front Nutr. 2025 Nov 7;12:1639068. doi: 10.3389/fnut.2025.1639068 (PMC12637025; doi:10.3389/fnut.2025.1639068)
Supplement: Supplementary file 2 [file Table_1.docx]

**Supplementary Table S1. Association of serum ferritin with HDP risk evaluated across prespecified subgroups.**

|  | SF with HDP risk in early pregnancy | | SF with HDP risk in late pregnancy | | |
| --- | --- | --- | --- | --- | --- |
| Subgroup | **Adjusted ^a^ OR (95% CI)** | **P for interaction ^b^** | **Adjusted ^a^ OR (95% CI)** | **P for interaction ^b^** | |
| Age(years) |  | 0.5085 |  | 0.5978 | |
| Age<35 | 1.014(0.999, 1.029) |  | 1.113(1.062,1.165) |  | |
| Age≥35 | 1.018(0.991,1.043) |  | 1.078(0.998-1.16) |  | |
| GDM(yes/no) |  | 0.5143 |  | 0.6129 | |
| Non GDM | 1.015(1.001,1.029) |  | 1.126(1.077, 1.176) |  | |
| GDM | 1.024(0.994,1.053) |  | 1.099(1.011, 1.194) |  | |
| Preterm(yes/no) |  | 0.9206 |  | | 0.2993 |
| Non preterm | 1.017(1.003,1.029) |  | 1.112(1.068,1.159) | |  |
| preterm | 1.020(0.974,1.065) |  | 1.165(1.032, 1.324) | |  |
| BMI(kg/m^2^) |  | 0.4969 |  | | 0.2084 |
| BMI<28 | 1.002(0.967,1.036) |  | 1.094(1.049, 1.140) | |  |
| BMI≥28 | 1.019(1.005,1.033) |  | 1.211(1.074, 1.382) | |  |
| Parity≥3(yes/no) | | 0.9019 |  | | 0.7069 |
| Parity<3 | 1.017(1.005,1.030) |  | 1.120(1.078, 1.165) | |  |
| parity≥3 | 0.936(0.701,1.159) |  | 0.879 ( 0.369,1.840) | |  |
| ICP(yes/no) |  | 0.8667 |  | | 0.5454 |
| Non ICP | 1.018(1.005, 1.030) |  | 1.120(1.077, 1.166) | |  |
| ICP | 0.999(0.897, 1.080) |  | 1.119(0.893, 1.377) | |  |
| PROM(yes/no) |  | 0.3281 |  | | 0.413 |
| Non PROM | 1.019(1.005,1.032) |  | 1.099(1.056, 1.144 ) | |  |
| PROM | 0.997(0.953,1.039) |  | 1.187(1.036, 1.338) | |  |

a Adjusted ORs were calculated per 10 μg/L increase in serum ferritin. Each stratification was adjusted for age(< 35 years old vs. ≥ 35 years old), GDM(yes/no), preterm(yes/no), BMI(< 28 vs. ≥ 28 kg/m^2^), parity(< 3 vs. ≥ 3), ICP(yes/no), PROM(yes/no). HDP: hypertensive disorders of pregnancy, OR: odds ratios.

b used the likelihood ratio test comparing models with and without an interaction term.

**Supplementary Table S2. The combined effect of serum ferritin concentrations during two different trimesters on HDP (excluded 19 cases of HDP with delivery before 34 GW).**

| **Groups^a^ of SF at 8.0-13.6 GW** | **Groups^a^ of SF at 29.0-31.6 GW** | **HDP(n,%)** | **Adjusted^b^ OR(95%CI)** | **P** |
| --- | --- | --- | --- | --- |
| 1(<31.5μg/L) | 1(<7.5μg/L) | 103(5.4%) | 1.043(0.817,1.332) | .735 |
|  | 2-3 (7.5-18.1μg/L) | 106(5.2%) | 0.908(0.711,1.159) | .439 |
|  | 4 (>18.1μg/L) | 37(8.0%) | 1.462(1.011,2.114) | **.043** |
| 2-3 (31.5-81.9μg/L) | 1(<7.5μg/L) | 117(5.4%) | 1.031(0.815,1.305) | .798 |
|  | 2-3 (7.5-18.1μg/L) | 249(5.3%) | 1(ref) |  |
|  | 4(>18.1μg/L) | 123(6.5%) | 1.228(0.974,1.548) | .082 |
| 4 ( >81.9μg/L) | 1(<7.5μg/L) | 15(3.5%) | 0.713(0.411,1.236) | .228 |
|  | 2-3 (7.5-18.1μg/L) | 117(6.1%) | 1.221(0.968,1.540) | .092 |
|  | 4 (>18.1μg/L) | 147(7.4%) | 1.348(1.080,1.682) | **.008** |

a Pregnant women were stratified into 9 groups based on quartiles of SF concentrations at two measurements (low, <25 percentile; intermediate, 25~75 percentile; and high, >75 percentile in two measurements). This created a 3×3 matrix, with the intermediate group from both measurements (25th-75th percentile in both assessments) serving as the reference group.

b Logistic regression adjusted for maternal age(continuous), BMI(continuous) and ICP (yes/no), GDM(yes/no), PROM(yes/no) , ICP(yes/no) and Preterm(yes/no).
